# Perceived Social Support on the Relationship Between ADD/ADHD and Both Anxious and Depressive Symptoms Among Canadian Adults

**DOI:** 10.1177/10870547221136227

**Published:** 2022-11-22

**Authors:** Ross D. Connolly, Allyson Lamont, David Speed

**Affiliations:** 1 Memorial University of Newfoundland, St. John’s, Canada; 2 University of New Brunswick, Saint John, Canada

**Keywords:** ADD/ADHD, social support, anxiety, depression, adult

## Abstract

**Objective:**

The primary goal of the present research was to examine whether the relationships that social support demonstrates with both anxiety and depression varied between adults with and without ADD/ADHD in a Canadian sample.

**Method:**

Data were obtained from the 2012 Canadian Community Health Survey–Mental Health (*N* ≥ 16,354). Presence of social support, diagnosis of generalized anxiety disorder (GAD), and experience of major depressive episodes (MDEs) were estimated in the self-report ADD/ADHD and non-ADD/ADHD groups.

**Results:**

Although social support was negatively associated with having GAD or experiencing an MDE, and self-report ADD/ADHD was positively associated with these outcomes. Presence of self-report ADD/ADHD did not significantly modify the relationships between social support and GAD or MDE.

**Conclusion:**

Social support may be a protective factor against symptoms of anxiety and depression in the general Canadian population, for adults with and without ADHD.

Attention deficit hyperactivity disorder (ADHD) is a neurodevelopmental psychiatric disorder defined by impaired levels of inattention and hyperactivity–impulsivity (Childress & Berry, 2012). Research has linked ADHD to various co-occurring mental disorders, such as anxiety, depression, and substance use disorders (Connolly et al., 2019). Although ADHD and other co-occurring psychiatric conditions are associated with adverse social outcomes (Harris-Lane et al., 2021), limited research has examined social support in the context of adulthood ADHD (Houtepen et al., 2019). Social support can be defined as “support accessible to an individual through social ties to other individuals, groups, and the larger community” (Lin et al., 1979, p. 109). Social support is associated with positive outcomes that include better physical and psychological health (Ozbay et al., 2007). However, obtaining social support can be difficult for individuals with ADHD (Bernardi et al., 2012).

The purpose of the present research was to determine how social support and ADHD predict rates of anxiety and depression in Canadians, and to compare the associations that social support demonstrates with anxiety and depression in ADHD and non-ADHD populations.

Historically, ADHD was considered a diagnosis of childhood, with the disorder being thought to resolve during adolescence or early adulthood (Hill & Schoener, 1996). However, it is now recognized that although the symptoms of hyperactivity and impulsivity may wane with increasing age (Biederman et al., 2010), symptoms of the disorder persist into adolescence/adulthood for two-thirds of those diagnosed in childhood (Sibley et al., 2017). The lifetime prevalence of ADHD among adults in Canada is 2.7% (Connolly et al., 2019), which is comparable with adult prevalence rates of ADHD found in other countries (Fayyad et al., 2007). As a result, the fifth edition of the Diagnostic and Statistical Manual of Mental Disorders (DSM-5; American Psychiatric Association [APA], 2013) includes a definition of ADHD that captures the nature of the disorder as experienced by older adolescents and adults.

ADHD is associated with negative outcomes across academic, social, neuropsychological, and affective domains (Lee et al., 2011). Adults with ADHD are more likely to have lower educational attainment than their non-ADHD peers (Gjervan et al., 2012), along with increased difficulty obtaining and retaining employment (Erskine et al., 2016). Adults with ADHD are also more likely to engage in novelty-seeking and risky behaviors (Young & Bramham, 2012), to experience higher levels of marital or family dysfunction, and to experience more problems with friends and in intimate relationships (Eakin et al., 2004). Further, it has consistently been observed that, relative to adults without ADHD, adults with ADHD experience higher levels of anxious and depressive disorders (Biederman et al., 2012; Friedrichs et al., 2012; Michielsen et al., 2012).

## ADD/ADHD and Social Support

Humans have a profound need to connect with others to gain a sense of belonging, and to manage their behaviors around establishing and maintaining social connections (Deci & Ryan, 2000; Stegar & Kashdan, 2009). Social support can come from an array of sources, such as from family, friends, teachers, community, or any social group with which one is affiliated (Vildósola et al., 2012). Social support can be the tangible assistance provided by others or perceived support based on an individual’s perception of the availability of adequate assistance or care when needed (Cheng, 1997; Yasin & Dzulkifli, 2010). Social support is thought to provide affective and instrumental resources that help individuals cope with adverse life experiences and that stimulate psychological well-being by acting as a protective factor against anxious and depressive disorders (Houtepen et al., 2019). Inversely, research shows that low social support is a predictor of psychological problems and is specifically associated with depression and anxiety (Meshi & Ellithorpe, 2021; Roohafza et al., 2014; Teoh & Rose, 2001).

Adults with ADHD are variably effective in modulating how they interact with others, which in turn can have a detrimental effect on their relationships with family, friends, and colleagues (Groen et al., 2018). As a result, individuals diagnosed with ADHD often report less social support than individuals without ADHD (Bernardi et al., 2012), which could affect the accessibility of external supports for coping with stressful experiences, such as anxious and depressive symptoms.

## ADHD and Anxiety and Depression

Although anxiety disorders, depressive disorders, and ADHD are distinct classes of disorders with different developmental trajectories across the lifespan, these disorders have high rates of comorbidity and share common symptom presentations (Adler et al., 2008; Kessler et al., 2006; Sobanski et al., 2007). Approximately 85% of adults with ADHD have at least one psychiatric comorbidity, and about 60% have at least two (Kessler et al., 2006). Additionally, the presence of an anxious or depressive disorder comorbidity in adult ADHD has been associated with additive clinical effects in the form of greater global impairment, poorer illness related outcomes, greater resistance to treatment, and increased costs of illness (Sobanski et al., 2007). One way to conceptualize the relationships between ADHD and anxiety, and ADHD and depression, can be as negative feedback loops where the symptoms of one disorder serve as input for the other which results in a worsening of symptom presentation over time. While anxious and depressive symptoms often occur comorbidly with ADHD, they are a direct result of ADHD and continue to exist by virtue of untreated symptoms of ADHD (Biederman et al., 2009; Feifel, 2007).

The magnitude of increase in generalized anxiety disorder diagnoses among individuals with ADHD is larger than among those without ADHD: the population-wide prevalence of an anxiety disorder among individuals with ADHD being approximately 47% (Kessler et al., 2006), compared to 11.6% among the general population (Statistics Canada, 2013). Among adults being clinically treated for ADHD, approximately 34% meet criteria for at least one anxiety disorder comorbid with ADHD (Krone, 2014). Further, anxiety disorders have been linked to executive dysfunction, which can exacerbate symptoms of ADHD by negatively impacting social functioning and psychological wellbeing; Vitello & Waslick, 2010).

In addition to the comorbid relationship between ADHD and anxiety, ADHD and depression are also highly comorbid. One Canadian study suggested that 31% of adults with ADHD reported a comorbid diagnosis of a major depressive disorder, compared to 12.5% of their non-ADHD peers (Hesson & Fowler, 2018). Adults with comorbid ADHD and depressive disorders experience an earlier onset of depression, more severe symptoms of depression, more frequent depressive episodes, and more suicide attempts (Babinski et al., 2020; Biederman et al., 2012; Fayyad et al., 2007; Friedrichs et al., 2012). Further, depression exacerbates symptoms of ADHD, manifesting as restlessness, psychomotor agitation, difficulty concentrating, increased distractibility, and decreased attention (Prince, 2015).

Individuals with comorbid ADHD and anxiety and/or depression experience a lower quality of life than those who have only anxiety or depression (McIntyre et al., 2010). Given that social support is a predictor of quality of life (Helgeson, 2003), it is not wholly surprising that individuals with ADHD report lower levels of social support than their non-ADHD peers (Mastoras et al., 2018; Rokeach & Wiener, 2020). Although the relationships between ADHD and anxiety and/or depression have been well established (Biederman et al., 2012; Hesson & Fowler, 2018; Michielsen et al., 2012), relatively few have considered the role of social support in these associations (Harris-Lane et al., 2021; Houtepen et al., 2019).

## The Current Study

There is little known regarding the role of social support as a protective factor on anxiety and depression among Canadian adults with a diagnosis of ADHD. Therefore, the present research had two goals: to determine how social support and ADHD predict rates of anxiety and depression in Canadians, and to determine whether the relationships that social support demonstrates with anxiety and depression are contingent on whether an individual has ADHD. It was hypothesized that:

**H1:** Based on previous research (Meshi & Ellithorpe, 2021; Roohafza et al., 2014)

we expect that higher scores for social support will predict lower scores of anxiety and depression

**H2:** Based on previous research (Hesson & Fowler, 2018) we expect that presence of ADHD will predict higher scores of anxiety and depression.
**H3:** Based on our assumptions in H1 and H2, we expect higher scores for social support will attenuate the association between symptoms of ADHD and symptoms of depression and anxiety.

## Method

### Data

All participants in the current study were respondents from the 2012 Canadian Community Health Survey—Mental Health (CCHS-MH) component (Statistics Canada, 2013, 2014). The CCHS-MH is a national survey collecting cross-sectional data on the general Canadian population regarding mental health status, psychopathology, functioning in relation to mental health, and related sociodemographic variables. Data for the survey were collected via a three-stage random cluster sampling design: geographical areas were divided into clusters and several clusters randomly selected; households were then randomly selected from each designated cluster; and one person aged 15 years or older was randomly selected from each household. Approximately 98% of the Canadian population is represented within the CCHS-MH and the response rate was 68.9%. Importantly, Statistics Canada’s sampling frame did not cover people living in territories, full-time members of the Canadian Armed Forces, people who are institutionalized, or peoples living in Aboriginal settlements.

In the survey database, age is recorded categorically and ranges from “15 to 19 years” to “80 years or older.” As the focus of the present study was on non-elderly adults, individuals in the age category “15 to 19 years” (*n* = 2,024) were not included in the current study. To allow for comparison with other prevalence studies (Connolly et al., 2019; Hesson & Fowler, 2015; Ramos-Quiroga et al., 2013), individuals aged 65 years and older were also not included in the analyses (*n* = 6,117). Additionally, CCHS-MH respondents must have answered all questions related to the covariates and predictors, as well as provided a valid response to at least one of the two outcomes. The minimum sample size for the current study was *N* = 16,354, which represented 20,427,374 Canadians (see Table 1 for descriptive statistics).

**Table 1.** Weighted Descriptive Statistics, Expressed as Either % or M/SD, for Respondents that Answered All Questions of Interest.

	All respondents	Non-ADHD	ADHD
	*N* = 16,223	*n* = 15,761	*n* = 462
Sex (% female)	50.1%	50.4%	36.6%
Region			
Atlantic	6.7%	6.8%	5.9%
Quebec	23.5%	23.2%	32.5%
Ontario	38.9%	39.1%	31.8%
Prairies	17.7%	17.7%	16.9%
British Columbia	13.2%	13.2%	12.8%^a^
Income	5.84/2.87	5.87/2.87	4.91/2.88
Education			
< High school	10.0%	9.8%	17.7%
High school	15.8%	15.7%	18.9%
Some post-sec.	6.4%	6.3%	9.9%^a^
Post-sec. grad	67.8%	68.2%	53.5%
Marital status			
Married/Common-law	65.7%	66.1%	52.2%
Wid./Sep./Div.	10.0%	10.0%	10.5%^a^
Single	24.3%	23.9%	37.3%
Race (% non-White)	24.5%	24.7%	16.1%^a^
Social Support	36.15/4.33	36.18/4.40	34.83/4.99
GAD (% yes)	2.8%	2.5%	13.0%^a^
MDE (% yes)	5.1%	4.7%	18.6%

*Note*. Cells may not total to 100% due to rounding; Post-sec. = post-secondary; Wid./Sep./Div. = widowed, separated, or divorced; GAD = generalized anxiety disorder; MDE = major depressive episode; ADHD = attention deficit hyperactivity disorder. Descriptive statistics for age cannot be presented due to a count violation based on Statistics Canada release guidelines.

a Indicates that the coefficient of variation for the cell was greater than 16.5%, but less than 33.3%, in following Statistics Canada release guidelines.

#### Sociodemographic variables

The present research controlled for a variety of covariates, including age (coded in 5-year increments from “20 to 24 years” to “60 to 64 years”), sex (0 = Female, 1 = Male), region (1 = Atlantic provinces, 2 = Quebec, 3 = Ontario, 4 = Prairies, 5 = British Columbia), decile for household income (measured continuously from 1 to 10), education level (1 = Less than high school, 2 = High school graduate, 3 = Some post-secondary, 4 = Post-secondary graduate), marital status (1 = Married/Common-law, 2 = Widowed/Separated/Divorced, 3 = Single), and race (0 = Non-White, 1 = White).

#### Social support

Perceived social support was measured using respondents’ scores on the Social Provisions Scale, a 10-item scale validated by Caron (1996; SPS-10) and adapted from the original 24-item Social Provisions Scale by Cutrona and Russell (1987). The SPS-10 is designed to measure five types of social support: attachment, guidance, integration, reliable alliance, and reassurance of worth (Orpana et al., 2019), using items such as “there are people I can depend on to help me if I really need it.” Each of the 10 items is rated on a four-point Likert scale ranging from 1 (S*trongly disagree*) to 4 (S*trongly agree*), and responses are summed to create the continuous scale. Possible scores range from 10 to 40, with greater scores indicating greater social support. The SPS-10 was found to be reliable in the current study (Cronbach’s α = .93).

#### Attention deficit (hyperactivity) disorder

CCHS-MH respondents were asked about whether they had been diagnosed with any mental health conditions that had persisted for, or were expected to persist for, 6 months or longer. In one question, respondents were asked, “Do you have attention deficit disorder?” which was used in the current study to classify whether they did or did not have ADHD. Responses were recoded as 0 (No) or 1 (Yes) in the current study. To build the interaction term, this variable was multiplied by the social support measure (i.e., ADHD × SPS10).

#### Anxiety

The 2012 CCHS-MH contained items from the World Health Organization’s Composite International Diagnostic Interview (WHO-CIDI), a standardized instrument used for the assessment of mental health disorders and conditions based on the definitions and criteria of the Diagnostic and Statistical Manual of Mental Disorders (DSM-IV). Several questions from the WHO-CIDI pertained to episodes of generalized anxiety disorder (GAD) in the previous 12 months. Experiencing GAD was defined as worrying about several different things or having diffuse worries (e.g., worried about “everything” or about “nothing in particular”) in the past 12 months. This definition was used to assess whether respondents met the criteria for experiencing GAD in the year prior to completing the CCHS-MH survey: responses were recoded as 0 (No) or 1 (Yes) in the current study.

#### Depression

Several of the questions from the WHO-CIDI from the CCHS-MH dataset also pertained to having experienced a major depressive episode (MDE) in the previous 12 months. An MDE was defined as a period of two or more weeks in the past 12 months in which an individual reported experiencing one of the three following categories of symptoms: feeling sad, empty, or depressed; losing interest in most things; or feeling discouraged about how things were going in their life. This definition was used to assess whether respondents met the criteria for experiencing an MDE in the year prior to completing the CCHS-MH survey: responses were recoded as 0 (No) or 1 (Yes) in the current study.

### Data Analysis

All data analysis was performed using Stata version 15 and all figures were made with Microsoft PowerPoint. A series of hierarchical binary logistic regression analyses were employed to examine the associations that ADHD, social support, and their respective interactions demonstrated with either GAD or MDE. All analyses were weighted with a person-level weight provided by Statistics Canada, meaning that estimates from the current study could be applied to Canadians who were between 20 and 64 years old and who were included in the sampling frame. Although logistic regression does not have an assumption of homoscedasticity, because of the non-simple random sampling approach of the CCHS-MH, HC1 corrections were applied for each coefficient estimate. Finally, tests for multicollinearity revealed no substantive issues and analyses proceeded as planned. Each model progressed in the same fashion, with two unique models being created for each of the two mental health outcomes:

Block 1: An outcome (GAD or MDE) was regressed onto covariates (Age, sex, region, household income, education level, marital status, and race).
Block 2: Social support was added to the model.
Block 3: ADHD was added to the model.
Block 4: The interaction term (social support × ADHD) was added to the model.

## Results

### Social Support, ADHD, and Anxiety

**Figure 1.**
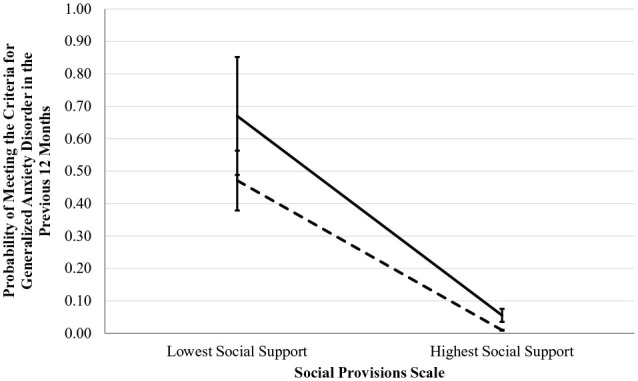
Differences in the prediction of anxiety in the previous 12 months based on social support, by presence of ADHD.

Covariates were entered in Block 1, *F*(20) = 6.03, *p* < .001. In Block 2, social support was entered, and was negatively associated with experiencing GAD in the previous year, *F*(1) = 116.28, *p* < .001. Every one-unit change in the SPS-10 score was associated with a decreased likelihood of having experienced anxiety, *OR* = 0.86, 95% CI [0.84, 0.88]. At this stage, differences were examined between respondents with the lowest score on the SPS-10 (i.e., a score of 10) and those with the highest score (i.e., 40): on average, respondents with the lowest recorded level of social support reported a 49.6% probability of experiencing GAD, compared to a 1.1% probability of GAD reported by respondents with the highest recorded level of social support (*M*_diff_ = −48.5%, *t* = −5.65, *p* < .001). In Block 3, ADHD was entered into the model, *F*(1) = 38.97, *p* < .001, and was positively associated with GAD, with a large effect size, *OR* = 4.72, 95% CI [2.90, 7.69]. To examine if the association between social support and experiencing GAD differed across individuals with and without ADHD, the interaction term (ADHD × SPS10) was entered in Block 4, though was not statistically significant, *F*(1) = 0.68, *p* = .411. See Table 2 and Figure 1 for details.

**Table 2.** Prediction of GAD in the Previous 12 Months Based on Covariates, Social Support, and ADHD, with ADHD as a Moderator.

*N* = 16,354	Odds ratio [95% Confidence Intervals]			
	Block 1	Block 2	Block 3	Block 4
Constant	0.04 [0.02, 0.08]***	5.19 [1.61, 16.72]**	3.97 [1.21, 12.98]*	4.45 [1.33, 14.91]*
Sex	0.67 [0.52, 0.87]**	0.57 [0.44, 0.74]***	0.54 [0.42, 0.71]***	0.54 [0.41, 0.70]***
Atlantic (ref.)				
Quebec	0.68 [0.46, 1.00]*	0.75 [0.51, 1.10]	0.72 [0.48, 1.06]^†^	0.71 [0.48, 1.05]^†^
Ontario	0.86 [0.60, 1.25]	0.80 [0.55, 1.15]	0.80 [0.55, 1.16]	0.80 [0.55, 1.16]
Prairies	0.99 [0.67, 1.45]	1.04 [0.70, 1.54]	1.03 [0.70, 1.53]	1.03 [0.70, 1.52]
British Columbia	1.00 [0.67, 1.47]	0.97 [0.65, 1.44]	0.96 [0.64, 1.45]	0.96 [0.64, 1.44]
Income	0.86 [0.81, 0.90]***	0.91 [0.87, 0.96]***	0.92 [0.87, 0.97]**	0.92 [0.87, 0.97]**
<High school (ref.)				
High school	0.76 [0.46, 1.27]	0.93 [0.56, 1.57]	0.95 [0.56, 1.59]	0.95 [0.57, 1.60]
Some post-sec.	0.82 [0.46, 1.44]	0.95 [0.53, 1.70]	0.96 [0.54, 1.73]	0.97 [0.55, 1.74]
Post-sec. grad	0.85 [0.56, 1.28]	1.11 [0.72, 1.71]	1.14 [0.74, 1.76]	1.15 [0.75, 1.77]
Married/Common-law (ref.)				
Wid./Sep./Div.	1.90 [1.33, 2.72]***	1.69 [1.17, 2.42]**	1.62 [1.12, 2.33]*	1.62 [1.13, 2.33]**
Single	1.63 [1.16, 2.29]**	1.35 [0.96, 1.90]^†^	1.35 [0.96, 1.89]^†^	1.35 [0.96, 1.88]^†^
White	1.83 [1.26, 2.67]**	2.15 [1.47, 3.15]***	2.04 [1.39, 3.01]***	2.04 [1.39, 2.99]***
Social Support		0.86 [0.84, 0.88]***	0.86 [0.84, 0.89]***	0.86 [0.84, 0.89]***
ADHD			4.72 [2.90, 7.69]***	1.65 [0.13, 20.23]
Social Support*ADHD				1.03 [0.96, 1.12]
Δ*F*	6.03***	116.28***	38.97***	0.68
Overall *F*-statistic	6.03***	12.45***	15.05***	14.24***

*Note.* Ref. = reference category for comparison; Post-sec. = post-secondary; Wid./Sep./Div. = widowed, separated, or divorced; ADHD = attention deficit hyperactivity disorder; Weighted population size for this sample = 20,427,374. Age was included in the regression models but excluded from the regression tables due to space constraints.

† *p* < .10. *p < .05. **p < .01. ***p < .001.

### Social Support, ADHD, and Depression

**Figure 2.**
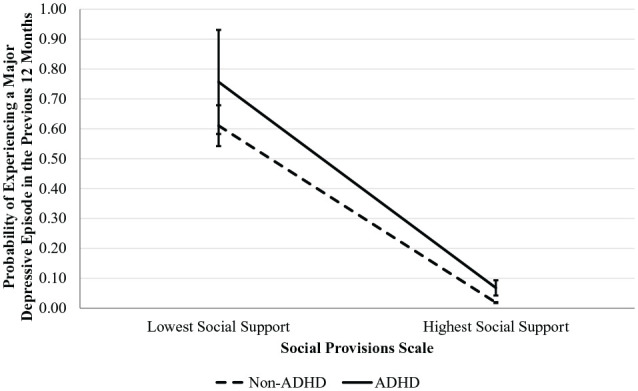
Differences in the Prediction of Experiencing a Major Depressive Episode in the Previous 12 Months Based on Social Support, by Presence of ADHD.

In Block 1, depression was regressed onto covariates, *F*(20) = 11.54, *p* < .001. In Block 2, social support was entered and was negatively associated with experiencing an MDE in the previous year, *F*(1) = 160.47, *p* < .001. Every one-unit increase in the SPS-10 was associated with a decrease in the probability of experiencing depression, *OR* = 0.86, 95% CI [0.84, 0.88]. Differences were again examined between respondents with the lowest score on the SPS-10 and those with the highest score: on average, respondents with the lowest recorded level of social support reported a 62.9% probability of experiencing an MDE, compared to the 2.0% probability of MDE reported by respondents with the highest recorded level of social support (*M*_diff_ = -60.9%, *t* = -9.01, *p* < .001). In Block 3, ADHD was entered, *F*(1) = 31.55, *p* < .001, and was positively associated with experiencing an MDE, and with a medium effect size, *OR* = 3.29, 95% CI [2.17, 4.99]. To examine if the association between social support and experiencing an MDE differed across individuals with and without ADHD, the interaction term between social support and ADHD was entered in Block 4, though was not statistically significant, *F*(1) = 0.26, *p* = .612. See Table 3 and Figure 2 for details.

**Table 3.** Prediction of an MDE in the Previous 12 Months Based on Covariates, Social Support, and ADHD, With ADHD as a Moderator.

*N* = 16,395	Odds Ratio [95% Confidence intervals]			
	Block 1	Block 2	Block 3	Block 4
Constant	0.13 [0.07, 0.22]**	17.45 [6.95, 43.82]**	14.65 [5.83, 36.82]**	15.53 [5.91, 40.8]**
Sex	0.70 [0.57, 0.86]*	0.61 [0.49, 0.75]**	0.58 [0.47, 0.72]**	0.58 [0.47, 0.72]**
Atlantic (ref.)				
Quebec	0.78 [0.57, 1.07]	0.86 [0.63, 1.18]	0.84 [0.61, 1.15]	0.83 [0.61, 1.15]
Ontario	1.05 [0.78, 1.43]	0.99 [0.72, 1.35]	0.99 [0.72, 1.36]	0.99 [0.72, 1.36]
Prairies	0.92 [0.66, 1.27]	0.96 [0.69, 1.34]	0.96 [0.68, 1.34]	0.95 [0.68, 1.34]
British Columbia	0.95 [0.68, 1.33]	0.91 [0.64, 1.29]	0.91 [0.64, 1.29]	0.91 [0.64, 1.29]
Income	0.85 [0.81, 0.88]**	0.89 [0.86, 0.93]**	0.90 [0.86, 0.94]**	0.90 [0.86, 0.94]**
<High school (ref.)				
High school	0.73 [0.50, 1.06]^†^	0.86 [0.58, 1.27]	0.88 [0.60, 1.29]	0.88 [0.60, 1.29]
Some post-sec.	1.07 [0.68, 1.68]	1.26 [0.79, 2.02]	1.28 [0.80, 2.05]	1.29 [0.81, 2.05]
Post-sec. grad	0.74 [0.54, 1.03]^†^	0.95 [0.67, 1.34]	0.98 [0.70, 1.37]	0.98 [0.70, 1.37]
Married/Common-law (ref.)				
Wid./Sep./Div.	1.90 [1.34, 2.70]**	1.71 [1.19, 2.46]*	1.67 [1.15, 2.42]*	1.67 [1.16, 2.42]*
Single	1.47 [1.15, 1.89]*	1.24 [0.96, 1.59]^†^	1.24 [0.96, 1.60]^†^	1.24 [0.96, 1.60]^†^
White	1.78 [1.33, 2.39]**	2.11 [1.56, 2.84]**	2.03 [1.49, 2.75]**	2.03 [1.50, 2.75]**
Social Support		0.86 [0.84, 0.88]**	0.86 [0.85, 0.88]**	0.86 [0.84, 0.88]**
ADHD			3.29 [2.17, 4.99]**	1.60 [0.11, 23.79]
Social Support × ADHD				1.02 [0.94, 1.11]
Δ*F*	11.54**	160.47**	31.55**	0.26
Overall *F*-statistic	11.54**	19.56**	21.46**	20.49**

*Note.* Ref. = reference category for comparison; Post-sec. = post-secondary; Wid./Sep./Div. = widowed, separated, or divorced; ADHD = attention deficit hyperactivity disorder; Weighted population size for this sample = 20,483,243. Age was included in the regression models but excluded from the regression tables due to space constraints.

† *p* < .10. *p < .01. **p < .001.

## Discussion

The purpose of the present research was to determine how social support and ADHD predicted rates of anxiety and depression among Canadian adults, and to also determine whether the relationship that social support had with anxiety and depression was contingent on whether an individual has ADHD. These research questions were examined using CCHS-MH data. Demographic covariates were included in the analyses to ensure that the observed effects were not accounted for by other related constructs and that the observed relationships between social support, anxiety or depression, and ADD/ADHD were present with the inclusion of these covariates. Consistent with the literature, we observed that higher scores for social support were associated with lower scores for anxiety and depression (Vildósola et al., 2012; Yasin & Dzulkifli, 2010), and that a self-reported ADD/ADHD diagnosis was positively associated with higher anxiety and depression scores (Yang et al., 2013). However, regarding our moderation research question, the relationships that social support demonstrated with anxiety and with depression were not contingent on whether an individual self-reported a diagnosis of ADD/ADHD.

### Anxiety

The present study assessed whether social support was associated with experiencing GAD in the previous year. Results suggested that social support acted as a protective factor, as greater social support was associated with a decreased likelihood of experiencing anxiety, which was supported by a 49.1% probability of experiencing GAD among those reporting low levels of social support, compared to a 1.1% probability of experiencing GAD among those with high levels of social support. This finding is consistent with prior research that has indicated a relationship between perceived social support and anxiety (Cohen et al., 1986).

Results also indicated that problem anxiety was positively associated with a self-reported diagnosis of ADD/ADHD, which was consistent with the literature (Adler et al., 2008; Kessler et al., 2006). In the current study, an individual reporting an ADD/ADHD diagnosis reported a 4.7 increase in the odds of GAD in the previous year than an individual who did not report an ADD/ADHD diagnosis. However, there was not a significant difference between individuals with and without ADHD in terms of the association between social support and experiencing GAD, suggesting parity in the relationship between social support and anxiety between ADD/ADHD and non-ADD/ADHD groups.

### Depression

In addition to anxiety, the present study also assessed whether social support was associated with experiencing a MDE in the previous year. Consistent with the literature (Cowan et al., 2008), results suggested that social support acted as a protective factor, as greater levels of social support were predictive of a decreased likelihood of experiencing depression, which was supported by a 62.9% probability of experiencing an MDE among those reporting low levels of social support, compared to a 2.0% probability experiencing an MDE among those reporting high levels of social support.

Results also highlighted that problem depression was positively associated with a self-reported diagnosis of ADD/ADHD, which was consistent with the literature (Jarrett & Ollendick, 2008). In the present research, an individual reporting an ADD/ADHD diagnosis reported 3.29 greater odds of experiencing an MDE in the previous year than an individual who did not report an ADD/ADHD diagnosis. However, there was not a significant difference between individuals with and without ADHD in terms of the association between social support and experiencing an MDE, suggesting parity in the relationship between social support and depression in ADD/ADHD and non-ADD/ADHD populations.

### Social Support

The findings of the present research show that increased levels of social support play a significant role in reducing the risk of experiencing anxiety and depression, and that ADHD is a risk factor for anxiety and depression. However, the results also suggest that the relationships that social support demonstrates with anxiety and with depression are not contingent on having an ADHD diagnosis. Therefore, these findings suggest that social support may act as a protective factor by reducing levels of anxiety and depression, regardless of whether a person has been diagnosed with ADHD. This point is subtle but important – the salutary role that social support has with both GAD and MDE is not restricted to whether an individual has an ADHD diagnosis.

### Limitations and Direction for Future Research

There are several limitations to the current study. First, ADD/ADHD was assessed using self-report data: not all CCHS-MH respondents may have had the opportunity to be diagnosed by a health professional. Therefore, without a diagnostic interview from the respondents, and without access to their medical files, it was not possible to confirm the accuracy of respondents’ self-reports. This lack of verification of ADD/ADHD diagnosis may have also affected the strength of the associations between self-reported ADD/ADHD and GAD or MDE as reported in the current study. Second, there was no information available in the CCHS-MH data to identify whether participants had received any form of treatment for ADD/ADHD: without treatment information, the researchers could not determine whether treatment mitigated the relationships between ADD/ADHD and GAD and/or MDE. Third, information regarding subtypes of ADD/ADHD was not assessed, but may have been relevant, given that previous studies have found differences in patterns of anxiety and depression between subtypes (Friedrichs et al., 2012; Pineiro-Dieguez et al., 2014; Sobanski et al., 2008). Fourth, mental health disorders that commonly cooccur with ADD/ADHD, anxiety, and/or depression were not measured in the present research study, and thus the omission of these comorbid disorders could account for some of the variability observed in the results. Finally, as the focus of the present research was adults aged 20 to 64 in a cross-sectional sample, so these findings cannot be generalized to adolescent or elderly populations, nor can they be interpreted causally.

Although the aforementioned limitations present some degree of ambiguity in interpreting the results, it is important to note the strengths of the current study, namely its nationally representative sample of Canadians. Although researchers excluded some groups from the analyses, the retained sample still offers a high degree of external validity that was only possible because the CCHS-MH database was accessible. Within this database, Canadians from every province between the age of 20 and 64 were selected in a representative fashion.

Future research would benefit from using a more comprehensive measure of ADD/ADHD that includes classification of symptoms and presentations, rather than relying on a self-reported diagnosis. Further, to address some of the limitations of the current study, future research should include additional comorbid mental health disorders as covariates in the data analyses, and should focus on adolescent and senior age groups. Another avenue for future research could include examining the role of negative social interactions in the relationships that an ADHD diagnosis demonstrates with anxiety and with depression among an adult population. Social problems are a common feature among children with ADHD (Cantwell, 1996; Friedman et al., 2003), but there is little known regarding the impact of negative social interactions across other age groups is limited, despite difficulties including social rejection and interpersonal relationship problems (Tseng & Gau, 2013).

## Conclusion

The current study investigated the relationships between anxiety or depression and social support in both the (self-report) ADD/ADHD population and the non-ADD/ADHD population within a Canadian context. Despite the limitations of the present research, the findings suggest that social support can act as a protective factor against anxiety and depression, although there was no significant difference in the effect of social support on mental health outcomes between the ADD/ADHD and non-ADD/ADHD groups.

Although it is not possible to “cure” ADHD, it is possible to alleviate its symptoms and to perhaps prevent the development of comorbidities such as anxiety and depression. It has been suggested that the sense of well-being reported by individuals receiving psychological treatment for ADHD is the result of relieving anhedonia and dysphoria (Mariani et al., 2014). The positive outcomes from therapy could in part be the result of positive social interactions that the client experiences in treatment. Strategies to bolster positive social interactions may include providing family psychoeducation and interpersonal skill training (Dour et al., 2014).

Further, research has shown that individuals with anxious and depressive disorders respond more strongly and positively than controls when they encounter positive events (Krone, 2014). The results from this research could support clinicians in encouraging clients to seek out positive social interactions. Additionally, clinicians could enhance positive interactions by being actively encouraging and supportive when engaging with clients to reinforce positive social interchange (Gable et al., 2004). These positive social interactions could reinforce the idea that the client is important to others by acting as a buffer against negative interactions that they frequently experience while also promoting health and wellness (Steese et al., 2006).
